# Balance strategy in hoverboard control

**DOI:** 10.1038/s41598-022-08291-0

**Published:** 2022-03-16

**Authors:** Mohammad Shushtari, Atsushi Takagi, Judy Lee, Etienne Burdet, Arash Arami

**Affiliations:** 1 Department of Mechanical and Mechatronics Engineering, University of Waterloo, Waterloo, ON N2L 3G1 Canada; 2 NTT Communication Science Laboratories, 3-1 Morinosato Wakamiya, Atsugi, Kanagawa 243-0198 Japan; 3 Department of Biomedical Engineering, Imperial College of Science, Technology and Medicine, London, UK; 4 Toronto Rehabilitation Institute (KITE), University Health Network, Toronto, ON Canada

**Keywords:** Biomedical engineering, Motor control

## Abstract

This study examines how people learn to perform lower limb control in a novel task with a hoverboard requiring to maintain dynamic balance. We designed an experiment to investigate the learning of hoverboard balance and two control strategies: A hip strategy, which mainly uses hip movements to change the angle of the foot, and an ankle strategy relying more on ankle motion to control the orientation of hoverboard plates controlling the motion. Motor learning was indicated by a significant $10\pm 4$% decrease in the trial completion time (p < 0.001) and a significant 24 ± 11% decrease in total muscle activation (p < 0.001). Furthermore, the participants, who had no prior experience riding a hoverboard, learned an ankle strategy to maintain their balance and control the hoverboard. This is supported by significantly stronger cross-correlation, phase synchrony, lower dynamic time warping distance between the hoverboard plate orientation controlling hoverboard motion, and the ankle angle when compared to the hip angle. The adopted ankle strategy was found to be robust to the foot orientation despite salient changes in muscle group activation patterns. Comparison with results of an experienced hoverboard rider confirmed that the first-time riders adopted an ankle strategy.

## Introduction

Balance control integrates sensory information from the visual, vestibular, and somatosensory systems^1–3^ and involves different spinal and supraspinal reflex circuits, reticulospinal descending tract, and cortical control of upper and lower limbs. From a biomechanical perspective, balance control is divided into static and dynamic categories. In static balance, equilibrium is maintained by modulating the center of pressure (COP) such that the body’s center of gravity (COG) is always kept within the base of support while in dynamic balance, like walking^4^, the COG may go outside of the base of support.

Dynamic balance is essential for locomotion and sports activities, such as snowboarding^5.^ It also plays an important role in the control of novel means of transportation like the hoverboard and Segway^6–8^. In contrast to the Segway, the hoverboard has no built-in stabilization. Instead, the stability comes from the rider, making the balance control more challenging in the initial phase of learning; however, it also makes the hoverboard more agile than the Segway as the COG can be shifted further away from the base of support. Balancing on the hoverboard resembles the balance control in quiet standing in the anterior/posterior direction where the feet are placed stationary side-by-side.

During quiet standing, people pivot about either the ankle or the hip to maintain their posture. The ankle strategy is discernible from dominant activation of muscles acting on the ankle resulting in plantarflexion and dorsiflexion to compensate for COG changes. The hip strategy involves the flexion and extension of the hip to regulate the COG to maintain balance. When the majority of the balancing comes from the hip, the ankle muscles show little agonistic activity^4,9^. Balance strategies are investigated by spatiotemporal analysis of the center of mass (COM) and joint angles when the balance is perturbed by a moving platform. In this regard, the relative phase and gain of the COM with respect to the platform displacement is used to distinguish elderly and young individuals’ balance strategy^10^ and to determine the potential of multiple sclerosis patients in adapting their balance strategies in the presence of random platform movements^11^. The same analysis is applied to the joint angles and showed that after balance training the balance strategy shifts from an ankle strategy to a multi-joint strategy to compensate for random platform motions^12^. Additionally, as balance requires postural control about an unstable equilibrium (standing is similar to stabilizing an inverted pendulum), it requires learning an appropriate strategy to modulate the mechanical impedance at different joints^13^, which can be achieved through selective muscle co-activation. To this end, several studies have looked at the mechanical impedance of the hip and ankle in static posture^14–16^ and during walking^17,18^.

Maintaining balance on a hoverboard is similar to stabilizing a cart-pendulum system^19^. Compared to quiet standing where the COG is kept within the base of support using ankle or hip strategies, the COP on the hoverboard is not directly controlled by the user. Instead, it is indirectly manipulated by the tilt angles of the hoverboard plates. As the user tilts the plates, the motors activate and rotate, generating a torque on the hoverboard’s wheel resulting in hoverboard translation. The user exploits this feature to perform goal-driven movements and to maintain balance, which is achieved by moving the base of support, a narrow area comprising the ground contact area of the two wheels that contains the COG. While balancing on a hoverboard or on the firm ground may appear to be very different in terms of control, in both cases, the COG can be controlled by dorsiflexion/plantarflexion of the ankle, by flexion/extension of the hip or a multi-joint strategy^12^. The feet also remain stationary during both hoverboard standing and quiet standing, and in both situations either the tilt position (in hoverboard riding) or the applied torque (in quiet standing) must be regulated to maintain balance. Due to these similarities, the same terms of the ankle and hip strategies for hoverboard balance control are used in this paper. Each of these strategies is defined by which joint contributes more dominantly to the control of the orientation of the hoverboard plates.

Riding a hoverboard and performing goal-driven movements is, nevertheless, more complicated than quiet standing. For instance, a hoverboard rider has numerous degrees of freedom in moving the hoverboard forward or backward (including variations in the velocity and the trajectory of the hoverboard). This could lead to a variety of strategies for performing the motion while maintaining balance. This makes the hoverboard control a challenging and interesting motor control task, in which we can analyze the lower limb motor learning in first-time riders and investigate if a general control strategy emerges and if a specific group of muscles would be recruited for this task. The specific aims of this study are to (1) investigate if early motor learning in a novel full-body motor task (with a strong role of lower limbs) appears only in task-relevant measures (e.g., trial elapsed time) or also in muscle recruitment (e.g., decrease in muscle activation or co-activation), and if specific metrics can be identified to explain better riding performance; (2) identify the adopted balance strategy, its indicators and their correlation with performance; (3) investigate the robustness of the adopted hoverboard control strategy with respect to the feet postural variations and if such variations can interfere with early motor learning.

## Methods

### Experimental setup

**Figure 1:**
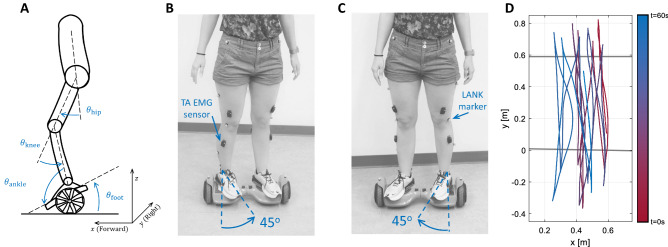
(**A**) Schematic of the joint angles and the hoverboard plate orientation (side view). (**B**) The feet are oriented 45$^\circ$ to the left of the hoverboard, corresponding to conditions A1 and A2. (**C**) Feet orientation in condition B. (**D**) Movement trajectory in the x-y plane from a representative participant #9 in condition A2. The line color (from red to blue) indicates the time. Grey lines indicate the goal lines participants had to cross.

A hoverboard (Bluefin Classic Scooter, UK) was used for the experiment, which is equipped with two 350 W brush-less DC motors and reaches a maximum speed of 16 km/h. A hoverboard can be modeled as a cart pendulum system where its movement is controlled by the applied tilt to its plates (Fig. 1A). Ten MX13+ Cameras (Vicon, UK) were used to capture the kinematics of the hoverboard and participants’ lower limb positions at 150 Hz. A setting of “PlugInGait” with 16 markers was used to calibrate and label the participant’s lower limb. Markers were placed on the left/right anterior superior iliac spine (LASI/RASI), left/right posterior superior iliac spine (LPSI/RPSI), the lower lateral 1/3 surface of the left/right thigh (LTHI/RTHI), the flexion-extension axis of the left/right knee (LKNE/RKNE), the lower 1/3 surface of the left/right shank (LTIB/RTIB), the lateral malleolus along an imaginary line that passes through the transmalleolar axis of left/right ankle (LANK/RANK), the calcaneus at the same height above the plantar surface of the left/right foot as the toe marker (LHEE/RHEE), and the second metatarsal head, on the mid-foot side of the equinus break between fore-foot and mid-foot of the left/right leg (LTOE/RTOE). Two additional markers were placed on the hoverboard extremities in order to measure its movement using motion capture.

Eight wireless electromyography (EMG) sensors (Trigno, Delsys, USA) with a sampling rate of 3000 Hz were placed on the following muscles of both legs: Tibialis Anterior (TA), Gastrocnemius Medial Head (GMH), Biceps Femoris (BF), and Rectus Femoris (RF). EMG sensors were placed on the skin after shaving and skin surface cleaning with ethanol. The motion capture system and EMG sensors were calibrated and synchronized.

### Experimental protocol

All participants provided written informed consent prior to participation. The study’s protocols and procedures were approved by the University of Waterloo (ORE#40451), Clinical Research Ethics Committee and conformed with the Declaration of Helsinki. Ten participants (age: $21.9\pm 0.74$ years, and five females) without known sensorimotor impairment and without previous experience in riding a hoverboard or similar self-balanced equipment were recruited. They went through a 5-min familiarization session with the hoverboard to learn how to step up and down from it, stabilize their balance, and move and turn with feet placed neutrally (straight on the hoverboard plates) without any specific instruction.An additional participant (Male, age 38 years) with prior experience of riding a hoverboard was also asked to perform the trials. His data, considered as the “expert” data, was used for comparison purposes.

The experiment consisted of back and forth movements with the hoverboard with two non-straight feet orientations. This would provide a novel, stable, and repeatable yet challenging task different than the familiarization phase. While back and forth movement reduces the required planning, the tilted feet orientation on hoverboard plates allows for a systematic study of the effect of feet postural variations on motor learning and balance strategy. Colored tape was used to draw two parallel lines (Bottom and Top line) on the ground with a distance of 0.6 m. Starting from the middle of the Bottom line, all participants were asked to perform as many back and forth movements between the two lines as possible (Fig. 1D). Forward movement is defined from the Bottom line to the Top line and backward movement from the Top to the Bottom line. One trial consists of a forward followed by a backward movement. The main experiment was split into trials under two different conditions. In the first condition (A1), participants were asked to stand on the hoverboard with their feet tilted at 45$^\circ$ to their left (Fig. 1B). In the second condition (B), they were asked to perform the same movement with their feet oriented at 45$^\circ$ to their right (Fig. 1C). Finally, participants completed movements with the feet oriented towards the left again (A2 condition). Each participant completed three conditions in total (A1, B, A2). Each condition lasted 60 s. Foot posture was checked before the start of each condition. After these trials, maximum voluntary contraction (MVC) of each muscle was also recorded to normalize the EMG signals^20^.

When the feet are oriented to the left in A1 condition, the right foot has a smaller moment arm compared to the left foot for applying torque to the hoverboard plate to move forward. The opposite is valid when feet are oriented to the right in condition B. Therefore, the dominant leg in the control of the hoverboard changes with the orientation of the feet. Assuming that a participant has learned a specific balance strategy in A1, condition B is introduced to interfere with their learned strategy and to test the robustness of the adopted strategy. The third condition (A2) tests whether the novel condition B had any impacts on the balancing strategy learned during the initial condition A1.

### Data analysis

#### Preprocessing

One of the participants failed to perform the trials, and was excluded from the analysis. The data from the motion capture system and EMG sensors were split into half-trials (forward/backward motion in each trial). This segmentation was carried out based on the extraction of prominent peaks of hoverboard position (computed on the data from the markers fixed on the hoverboard) smoothed using a moving average filter with a 0.67-s window. EMG signals were filtered with a third-order Butterworth bandpass filter with cutoff frequencies of 30 Hz and 450 Hz, respectively. After rectification, their RMS values were computed using a moving RMS filter with a 1-s window. Moving window lengths are tuned such that the data from different half trials are not blended while the high-frequency fluctuations are filtered out. Next, each signal was normalized to its corresponding MVC value. Finally, each muscle onset/offset was computed using thresholds defined by two standard deviations of EMG-baseline variations^20^ (See Fig. 3 for an example of raw and preprocessed data).

#### Hoverboard control performance analysis

We considered the number of trials at each condition and trial elapsed time, defined as the time taken to complete one trial, as the task performance measures since participants were asked to perform as many trials as they could in a minute. We used a Linear Mixed Model (LMM) to analyze the trial elapsed time and its reduction as a sign of motor learning in participants. We included the condition (A1, B, and A2) and movement direction (forward and backward) as fixed-effect intercepts, and treated the trial number as a fixed slope. To account for the correlation of repeated measurements for each participant, a random intercept and slope with respect to the trial number were also considered. Thus, the trial elapsed time ($T_{im}$) for a given observation *i* on participant *m* is modeled as

1 $$T_{{im}} = \beta _{0} + \beta _{1} {\text{B}}_{i} + \beta _{2} {\text{A}}2_{i} + \beta _{3} {\text{backward}}_{i} + \beta _{4} {\text{trial}}_{i} + b_{{0m}} {\text{sub}}_{{im}} + b_{{1m}} ({\text{trial}}_{{im}} ,{\text{sub}}_{{im}} ) + \varepsilon _{{im}}$$

where $\beta _0$, $\beta _1$, ...$\beta _4$ are the weights of fixed effects, while $b _{0m}$ and $b _{1m}$ are weights of random effects. The indicator variables $\text {B}_i$, $\text {A2}_i$, and $\text {backward}_i$ were set to one if observation *i* belonged to the respective condition or direction, otherwise they were set to zero. The slope variable $\text {trial}_i$ was also equal to the trial number. Similarly, $\text {trial}_{im}$ and $\text {sub}_{im}$ were set to the trial number and one, respectively, in case the observation *i* belonged to the participant *m*, otherwise were set to zero. $\epsilon _{im}$ captures the difference between the measured values ($T _{im}$) and the prediction of the model for participant *m*.The average relative change in trial elapsed time ($\Delta T_m$) in each condition is then computed for participant *m* based on the average number of performed trials $N_{\text {avg},m}$ in a condition and the average trial time ($T_{\text {avg},m}$) as

2 $$\begin{aligned} \small \Delta T_m=\frac{(\beta _4+b_{1m})\times N_{\text {avg},m}}{T_{\text {avg},m}}\times 100 . \end{aligned}$$

This measure shows how much the trial elapsed time has decreased on average during a condition compared to its average value. For example, if the average trial elapsed time of a participant is 5 s, a $\Delta T$ of $-10$% shows that the trial elapsed time has decreased by 0.5 s during each condition on average. We use this formula to convert the LMM estimations to relative average changes for a more tangible representation of the results.

#### EMG signal processing

The muscle co-activation between muscles *p* and *q* in the trial *i* ($C_{pq}^i$), was computed as:

3 $$\begin{aligned} C_{pq}^i = \frac{{\int _{t_{\text {start}}^i}^{t_{\text {end}}^i} {{e_p}\left( t \right)~{e_q}\left( t \right)~dt} }}{{t_{\text {end}}^i - t_{\text {start}}^i}} \end{aligned}$$

where $e_p$ and $e_q$ are the filtered, normalized EMG signals of muscles *p* and *q*, respectively. Setting $p=q$, we can compute the mean square muscle activation for each muscle. Among six different combinations of muscles for co-activation calculation, only three of them are interpreted: $C_{12}$ between Rectus Femoris and Gastrocnemius Medial Head, $C_{13}$ between Bicep Femoris and Rectus Femoris, and $C_{24}$ between Tibialis Anterior and Gastrocnemius Medial Head. $C_{12}$ and $C_{13}$ are related to the muscles that exert opposing torques at the knee joint. An increase in $C_{12}$ and $C_{13}$, referred from henceforth as knee co-activation type 1 and 2, respectively, would increase the knee joint mechanical impedance. Similarly, we call $C_{24}$ the ankle co-activation since it is related to the muscles that exert opposing torques on the ankle joint. $C_{11}$, $C_{22}$, $C_{33}$, and $C_{44}$ are also the muscle activation of Bicep Femoris, Gastrocnemius Medial Head, Rectus Femoris, and Tibialis anterior, respectively. The total muscle activation was also computed as $C_{\text{{Total}}}=\sum \nolimits _{j = 1}^4 {C_{jj}}$. Furthermore, to evaluate the effort and interaction of muscle activation and obtained speed during trial number *i*, we defined and used the measure

$$\begin{aligned} W_i = \int _{{t_{{\text {start}}}^i}}^{{t_{{\text {end}}}^i}} {\left( {\sum \limits _{p = 1}^4 {{e_p(t)}} } \right)~ .~\left| {{v_{{\text {hb}}}}(t)} \right|~ dt} \end{aligned}$$

where $v_\text {hb}(t)$ is the hoverboard speed. *W* has a unit of energy if $e_p$ is calibrated to the force. *W* can approximate the energy of the system, and is an upper bound for the work as the $e_p$ forces are not in the direction of resultant $v_\text {hb}(t)$.

We fitted an LMM to each of muscle activation ($C_{11},\dots ,C_{44}$), co-activation ($C_{12}$, $C_{13}$, and $C_{24}$), the total muscle activation ($C_\text {Total}$), and the system energy (*W*) to investigate motor learning. This model includes condition, leg, and movement direction as fixed intercepts. The interaction between condition and leg is also considered in the model in addition to a fixed slope for the trial number. Similar to the trial elapsed time model (Eq. 1), we considered random intercept and slope for capturing the correlation of measurements for each participant.

We also investigated the variations of total muscle activation with respect to each leg and condition. To focus on the mean value of the total activation in each condition and leg, we included only intercepts in the model. Fixed intercepts were considered for condition, leg and their interaction. Similar random effects were also considered to account for the correlation of repeated measurements for participants. Moreover, the average relative change in the system energy ($\Delta W_m$), muscle activation ($\Delta C^{m}$), and the total muscle activation ($\Delta C^{m}_{\text {Total}}$) are computed similar to Eq. (2).

#### Balance strategy analysis

To investigate how the hip and ankle balance strategies were adopted to control the hoverboard, the similarity between the hip and the ankle joints’ motion and the tilt angle of the hoverboard’s plates was evaluated (see Fig. 1A for the definition of angles). First, a range normalization was applied to all the trajectories within each condition due to different ranges of motion in the ankle, hip, and hoverboard plate angle (same as the foot angle). This was done by removing the mean value of each signal and dividing it by its maximum absolute value. Hoverboard motion is controlled with its plates orientation which is reflected in highly correlated hoverboard position in the y direction. We expect certain joint rotations contribute to these oscillations more dominantly according to the balance strategy. In contrast to studies that investigated the balance strategy in quiet standing using a 1-DOF independent moving platform^11,12^, we cannot use simple phase difference and gain analysis since the balance on the hoverboard is the outcome of the concurrent control of the hoverboard plates’ orientation and the hoverboard acceleration, which are controlled to maintain the balance and to perform back and forth motions. In addition, riding a hoverboard does not follow a fixed-frequency motion and depending on the intention of the rider, the hoverboard plates will move in a wide range of frequencies and possibly with variable time lags with respect to the joint angles. Hence, we used different similarity measures to account for the variability induced by the complexity of our task. To determine which joint has the greatest contribution to the control of the hoverboard plates’ orientation, three metrics were used, (1) maximum cross-correlation between joint angles and hoverboard plate orientation, (2) phase difference between signals, (3) the distance between the signals using Dynamic Time Warping (DTW).

**Figure 2:**
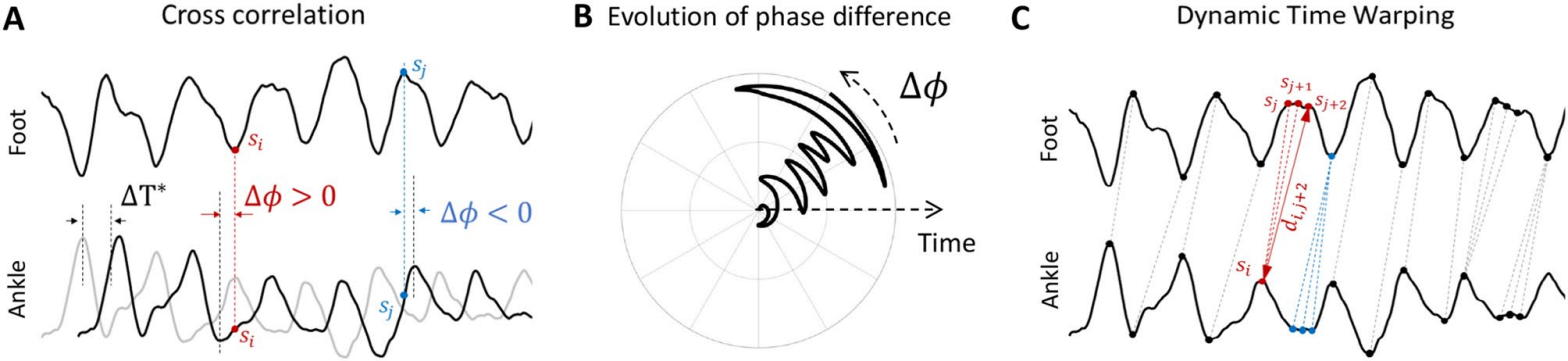
(**A**) Aligning foot and ankle joint angle profiles by shifting the original ankle angle (gray line) by $\Delta T^*$ to obtain maximum cross-correlation. The peaks of the ankle angle profile are not perfectly aligned. For example, at sample $s_i$ shifted ankle angle is leading while at sample $s_j$ it is lagging behind the foot orientation. (**B**) An example of the instantaneous phase difference variation between the ankle and the foot angles with respect to time. (**C)** Aligning signal samples using dynamic time warping. In this method, instead of shifting the whole ankle angle by a constant value, samples of the ankle joint profile are mapped to the samples of the foot profile. For example, sample $s_i$, representing a peak in ankle angle, is mapped to samples $s_j$ to $s_{j+2}$ since all those samples are representing the corresponding peak at the foot angle profile. The mapping is not injective and can thus account for variable delays between the signals.

The maximum cross-correlation between the hip and ankle angles with the foot angle was computed by shifting each of the ankle and hip angle profiles by their corresponding optimum lags ($\Delta T^*$). The use of cross-correlation as the only similarity measure can be debatable due to the complexity of the task as the delay between angle profiles is not fixed over a condition, and therefore, using a fixed lag value would not be reliable for revealing the synchrony (Fig. 2A). Furthermore, the correlations of the ankle and the hip angles with foot tilt are computed at different optimum lags which can add uncertainty to the cross-correlation comparison. To address these limitations, we used the mentioned two other metrics since they account for time-varying lag between signals.

The instantaneous phase^21^ difference between the foot orientation and the ankle and the hip angles are estimated by means of Hilbert transform^22^. We checked if there was any constant phase difference between the signals, which is a sign of synchronization between their oscillations. Figure 2B shows a visual description of foot-ankle instantaneous phase difference evolution. To compare the phase difference of hip and ankle joints with respect to the foot orientation, we fitted separate LMMs to each of them. We assumed that the phase slope with respect to time indicates the synchrony of the joints with foot orientation. A significant slope indicates an asynchronous motion while a zero (or non-significant) slope indicates synchrony. We included the condition and leg as fixed effect intercepts. Sample time ($\text {t}_i$) is treated as a fixed slope. The measured values ($\phi _{im}$) for a given observation *i* on participant *m* is modeled as

4 $$\phi _{{im}} = \beta _{0} + \beta _{1} {\text{B}}_{i} + \beta _{2} {\text{A2}}_{i} + \beta _{3} {\text{left}}_{i} + \beta _{4} {\text{t}}_{i} + b_{{0m}} {\text{sub}}_{{im}} + b_{{1m}} ({\text{t}}_{{im}} ,{\text{sub}}_{{im}} ) + \varepsilon _{{im}} .$$

A dynamic time warping (DTW) similarity measure^23^, which computes the spatial distance between signals regardless of their temporal differences, was also used to discriminate between the two balancing strategies. Since DTW is insensitive to shifts in the time domain, it is robust to phase shifts between two signals (see Fig. 2C). The DTW was computed between the foot orientation and the ankle or the hip angle. To track the changes of distance between the signals, we computed DTW locally within a moving window with length and steps of 33.34 s and 0.67 s, respectively. We fitted separate LMMs to each of the ankle and hip DTW data in order to compare each joint’s similarity to the foot orientation. Hence, the observation *i* for each computed distance (*d*) for the participant *m* is modeled as

5 $$d_{{im}} = \beta _{0} + \beta _{1} {\text{B}}_{i} + \beta _{2} {\text{A2}}_{i} + b_{{0m}} {\text{sub}}_{{im}} + b_{{1m}} ({\text{B}}_{i} ,{\text{sub}}_{{im}} ) + b_{{2m}} ({\text{A2}}_{i} ,{\text{sub}}_{{im}} ) + \varepsilon _{{im}}$$

where $d_{im}$ is the local distance between the observation *i* of hip or ankle joint from the foot orientation.

We adjusted the complexity of the LMMs by performing Log-likelihood Ratio Test and Akaike Information Criterion. The significance of each effect on the model was also tested using F-test at the 5$\%$ significance level.

## Results

**Figure 3:**
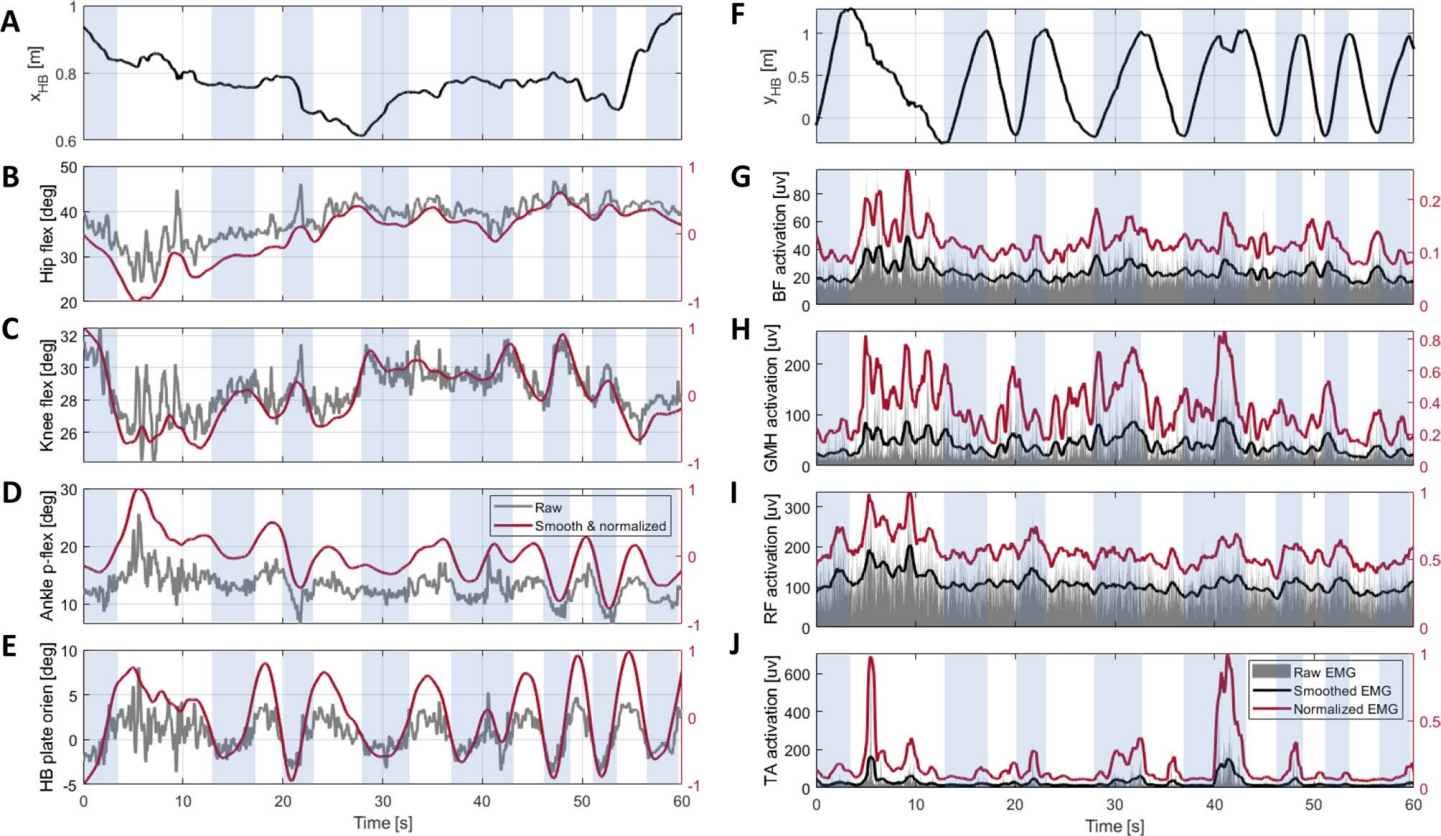
A typical raw and normalized time series of hoverboard centre position in x (**A**) and y (**F**) directions; (**B**) The hip, (**C**) knee, and (**D**) ankle angles; (**E**) the hoveboard plate orientation; (**G**) Bicep Femoris, (**H**) Gastrocnemius Medial Head, (**I**) Rectus Femoris, and (**J**) Tibialis Anterior muscle activation of the right leg in condition B for an average participant (#9). The blue area denotes the forward motion intervals. Value of the normalized signals are reported on the right axis of each figure.

Figure 3 shows the raw and the preprocessed kinematic data, including joint angles, foot orientation, and hoverboard position, and muscle activation in condition B for a representative participant #9 with an average performance according to Table 1. A similarity can be seen between the hoverboard plate orientation (Fig. 3E) and ankle plantar flexion angle (Fig. 3D). For this participant, in condition B forward motion half trials, GMH (Fig. 3H) and RF (Fig. 3I) exhibit higher activities, while in backward motions the muscles are relatively less active.

**Table 1:** Number of completed trials at each condition. Participants are sorted based on the total performed trials.

Participant #	7	4	8	10	9	6	1	2	5	Expert
A1	9	7	10	7	4	6	4	5	2	19
B	9	10	8	8	7	6	6	4	3	16
A2	11	12	9	8	9	6	5	5	4	19
Total Trials	29	29	27	23	20	18	15	14	9	54

### Motor performance and motor learning

We computed the performance of each participant by summing the number of completed trials in each condition (Table 1). We also evaluated the motor learning progress of each participant during each condition using the trial elapsed time and the total muscle activation. The system energy (W) is also considered as a measure of motor learning to capture the interaction effect of trial completion time and muscle effort.

**Figure 4:**
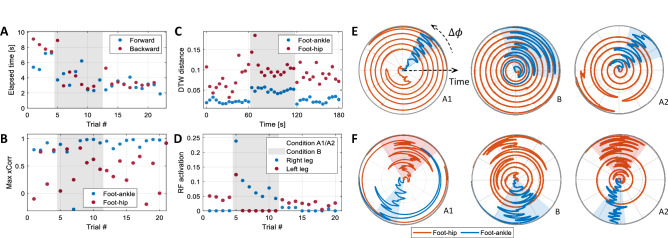
Results of a representative participant (#9): (**A**) Trial elapsed time in forward and backward motion; (**B**) Maximum cross-correlation between the foot, and the ankle and hip angles for the right leg in each trial; (**C**) DTW distance between the right foot angle, and the ankle and hip angles; (**D**) Rectus Femoris activation in backward movement in each trial for the right leg; (**E**) The instantaneous phase difference ($\Delta \phi$) between the right foot profile, and the right ankle and hip angles for A1, B, and A2 conditions. The distance from the origin represents the sample time while the angle represents the phase difference ($\Delta \phi$). In all conditions, $\Delta \phi _{\text {hip}}$ travels the whole phase circle several times. The $\Delta \phi _{\text {ankle}}$. (**F**) Phase analysis for the expert participant who tried to control the hoverboard mostly with the hip. The ankle phase is still bounded in all conditions similar to the ankle strategy. The hip phase is also bounded in conditions A1 and A2 and has fewer variations even in condition B as a result of adopting hip or a multi-joint strategy.

**Figure 5:**
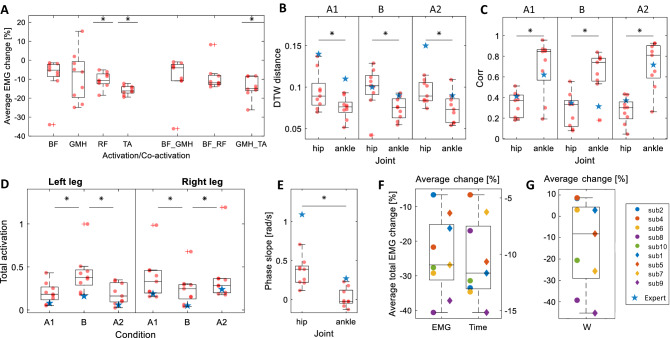
(**A**) Average relative change ($\Delta C^{m}$) for each activation and co-activation across participants. It shows the average reduction per condition relative to the average value. For example, if the mean activation of a muscle is 0.5, a $\Delta C$ of $-10$% means that its activation has dropped about 0.05 in each condition on average. (**B**) Comparison of average estimated values of the DTW distance for the hip and ankle joints from the foot angle across participants in three conditions. (**C**) Comparison of foot and the ankle and hip joints maximum cross-correlation across participants in three conditions. (**D**) Average total muscle activation in each condition and leg across participants. (**E**) Comparison of estimated phase slopes for the hip and ankle joints (the rate of phase change over sample time) across participants. (**F**) Comparison of the average relative change for trial elapse time ($\Delta T_m$) and the total muscle activation ($\Delta C^m_{\text {Total}}$) across participants. (**G**) average relative change of system energy ($\Delta W_m$) across participants.

Figure 4A,D illustrate the trial elapsed time and the Rectus Femoris activity for participant #9. Overall, a decrease in trial elapsed time and muscle activity can be observed as this participant performs more trials. In particular, Rectus Femoris muscle activity had a decreasing trend in the left leg during backward motion in condition B. To investigate the variations of motor learning indicators as a function of trial number, we fitted LMMs to the trial elapsed time and total muscle activation data, respectively. Both the trial elapsed time and the total muscle activation yielded significant negative slopes as a function of the trial number (F-test, $p_{time}<0.001$, $p_{Total EMG}<0.001$). Figure 5F illustrates the average trial completion time change and the average total muscle activation change for each participant across conditions computed based on the values estimated by corresponding LMMs. We then fitted an LMM to the individual muscle activation and co-activation data. Figure 5A shows negative average relative changes for most muscles ($\Delta C^m$). Although the estimated slope is not significantly different from zero in the activity of each muscle and co-activation of muscle pairs, an overall decreasing trend in muscle activity is observed, indicative of motor learning. In particular, the ankle joint co-activation has a negative average relative change (F-test, $p_{GMH\_TA}$ = 0.0092), signifying motor learning has occurred. Finally, we fitted an LLM model to the system energy to investigate the joint effect of muscle activation and trial elapsed time (Fig. 5G). Participants showed a 13 ± 19% reduction in system energy. That trend was not, however, found to be statistically significant (F-test, p = 0.2528). Comparing the number of completed trials, the elapsed time, and muscle activation time series across A–B–A conditions, we did not observe any sign of learning interference due to condition B.

### Balance strategy

As described, hoverboard balance is achieved by controlling the tilt angle of its plates. Oscillatory trajectories for the plates are to be expected during the forward and backward movements. This oscillatory behavior is not, however, equally mapped into each joint but is reflected mainly based on the adopted balance strategy. In other words, the foot orientation is simultaneously affected by the ankle, knee, and hip angles, so there is a multitude of ways to control the foot orientation.

#### Maximum cross-correlation

To investigate the contribution of the ankle and hip movements to the hoverboard tilt, we initially examined the maximum cross-correlation between the foot orientation and the ankle or hip angle profiles (see Figs. 4B and 5C). It was observed that the ankle joint delay from the foot orientation has less variation (0.7 ± 1.2 s) while the hip delay from foot orientation has a much larger standard deviation ($-0.2 \pm 3.4$ s). Results show significantly higher cross-correlation between the ankle and foot angles (0.69 ± 0.27, 0.63 ± 0.21, 0.71 ± 0.22, in conditions A1, B and A2, respectively) compared to the hip and foot angles cross-correlation (0.3 ± 0.1, 0.28 ± 0.17, 0.26 ± 0.12, in conditions A1, B and A2, respectively, F-test, $p_{A1} = 0.018$, $p_{B}=0.002$, $p_{A_2}<0.001$) indicating an ankle strategy being adopted.

For a comparison with possible hip strategy, we performed the same cross-correlation analysis on an experienced hoverboard rider when being asked to dominantly use their hip (simulated hip strategy) or ankle (simulated ankle strategy) during the A1 condition. During simulated ankle strategy, a maximum cross-correlation of 0.63 and 0.42 were obtained for ankle–foot angle and hip–foot angle, respectively. While the ankle–foot angle maximum cross-correlation remained almost the same (0.64) for the simulated hip strategy, we observed a remarkable increase of hip–foot angle maximum cross-correlation (0.69).

#### Phase difference

Figure 4E shows the phase difference for participant #9. The phase angle between the hip and the foot varies along with a wider range between 0 and $2\pi$ compared to the phase between the ankle and the foot, suggesting a stronger phase synchrony and the use of an ankle balancing strategy in conditions A1, B and A2. To determine whether other riders predominantly employed an ankle balancing strategy, we fitted the LMM in Eq. (4) to each of the ankle and hip joint phase difference data. Figure 5E shows the phase slope for the hip and ankle joints estimated by their corresponding models where the value of each point in the graph is equal to $\beta _4+b_{1m}$. The phase slope of the hip joint is 15 times larger than the ankle joint and according to the performed F-test on both models, the slope fix effect does not significantly contribute to the ankle model (F-test, $p_{\beta _4,{\text{ankle}}}~=~0.5706$) while the opposite applies to the hip model where there exists a significant positive fix slope (F-test, $p_{\beta _4,\text{hip}}<0.001$). These observations show that a small phase difference with the foot angle is maintained at the ankle while the hip joint has not maintained such a bounded phase difference with the foot angle. Therefore, the ankle joint moves in synchrony with the hoverboard’s plates, indicative of an ankle balancing strategy.

We also asked the expert participant to perform a similar goal-directed movement in the three conditions. We found that the expert has a higher phase slope in hip angle compared to the ankle angle. The contrast between the phase differences is greater compared to the experiment participants, indicating strong adoption of ankle strategy by the expert (see Fig. 5E). We then asked the expert participant to perform the same experiment, this time by using the hip joint mostly. Phase analysis of this scenario is illustrated in Fig. 4F. In contrast to the phase analysis of the ankle strategy (Fig. 4E), the hip strategy is emerged by smaller variations in the hip–foot phase differences while maintaining the same amount of ankle–foot phase variation.

#### DTW distance

The Dynamic Time Warping distance was used to measure the distance between the ankle and hip angles and foot orientation. The DTW distance from a sample participant #9 is shown in Fig. 4C. The DTW distance is smaller between the ankle and foot compared with the foot and the hip in all three conditions.

We fitted separate LMMs to the DTW data based on Eq. (5). Figure 5B shows the overall distance of each of the ankle and hip joint angles from the hoverboard plates’ orientation. The results show that the hip joint angle has a significantly larger DTW distance from the foot angle in comparison to the ankle angles by 26%, 32%, and 29% in A1, B, and A2 conditions (F-test, $p_{A1}=0.009$, $p_B=0.003$, $p_{A2}<0.0001$), respectively. DTW analysis of the data collected from the expert subject confirms the same conclusion. This indicates that participants used their ankles more than their hips in controlling the hoverboard plates.

#### Muscle activation

This subsection investigates how the task condition, i.e., *A1*–*B*–*A2* blocks with different foot orientations on the hoverboard, affected the muscle activity patterns and if condition *B* interferes with the balance strategy learned in *A1* at the muscular level. In the fitted LMM model to the activation and co-activation data, all of the fixed effects significantly contribute to the prediction of the response variable, but the predictor distinguishing between condition A1 and A2. This suggests that participants employed the same balancing strategy learned in A1 during A2. For further analysis, we computed the total muscle activation and fitted another LMM model to the total activation data. Figure 5D shows the mean value for each participant’s total activation in different conditions and legs. The estimated total activation in condition B is significantly different from conditions A1 and A2 for both legs (F-test, $p_{A1R,BR}=0.0088$, $p_{A2R,BR}=0.0294$, $p_{A1L,BL}=0.0074$, $p_{A2L,BL}=0.0047$), while conditions A1 and A2 do not have a significant difference in activation/co-activation (F-test, $p_{A1R,A2R}=0.3665$, $p_{A1L,A2L}=0.7050$), implying a difference in the strategy used in conditions A1/A2 and condition B. Total muscle activation data collected from the expert participant also confirms the same pattern.

## Discussion

### Motor learning

We observed in Fig. 5F that the time taken to complete one trial decreased by $10.56\pm 4.10\%$ (mean±SEM). Does this improvement come at the cost of increased effort? The system energy (analyzed using W, which is an upper bound for mechanical work) did not increase in the majority of our participants (Fig. 5G, the energy decreased by 10–45% in six participants, and increased by 5–10% in 3 participants), and muscle activation decreased across all subjects by $24.23\pm 11.26\%$. Co-activation is further intrinsic indicator that is larger at the early stages of learning especially in unstable tasks like balancing^24^. Large co-activation enables the motor system to keep the movement trajectory close to the planned one until the feedforward control of muscle activation is learned, gradually replacing the high feedback gains^25^. Our hoverboard riders initially had large co-activation in the ankle joint that decreased with the trial number, eventually lowering by $14.20\pm 5.68\%$ (Fig. 5A). A limitation of this study concerning the motor learning of hoverboard riding is that participants were not totally naive to the hoverboard at the beginning of the experiment as they received some practice during the familiarization phase. However, this could not be avoided as the rider’s safety was of primary concern. We could not ask participants who had never ridden a hoverboard to balance on it whilst moving forwards and backwards rapidly. While the analysis of motor learning during the familiarization phase has been omitted in our study, the reduction in the co-activation during the main experiment suggests that the motor learning was not complete prior to the experiment. Having the familiarization phase also helped us reduce the effect of fear and consequent muscle reflexes which could affect the total activation measure. Frequency analysis also confirms negligible fatigue during the experiment. Therefore, we considered the decrease of muscle activation to be mainly due to motor learning.

### Balance strategy

Maximum cross-correlation, phase difference evaluation and DTW distance analysis all suggest the adoption of an ankle strategy (dominant ankle role in the control of hoverboard). Analysis of the data collected from the expert participant shows similar patterns as those observed in the data collected from experiment participants in terms of maximum cross-correlation, phase slope, DTW, and muscle activation all of which indicate the use of the ankle strategy. When the expert participant utilized a hip strategy, we observed an increased maximum cross-correlation between hip angle and foot angle, and a reduced variation of hip–foot phase difference. Control of the ankle–foot phase difference might be essential for efficient control of the hoverboard regardless of the balance strategy judging by this expert’s data.

### Robustness of adopted balance strategy

An A–B–A paradigm was used in this study to investigate if the adoption of a balance strategy depends on the participants’ initial posture, and to verify the robustness of the learned motor control when asked to balance on the hoverboard using a different foot posture (condition B where the feet pointed rightwards, not leftwards as in conditions A1 and A2). According to Fig. 5D, the total muscle activation in condition B is different from the A1 and A2. Participants also found it difficult to perform trials at the beginning of condition B, as indicated by the increased elapsed time and curved spatial trajectories of the hoverboard. These results suggest that condition B required a riding strategy different from conditions A1 and A2. However, the balancing strategy of using the ankles to control the foot angle (or the tilt in the hoverboard plates) remained robust across conditions as the phase difference between the ankle and the foot was always smaller than between the hip and the foot.

**Figure 6:**
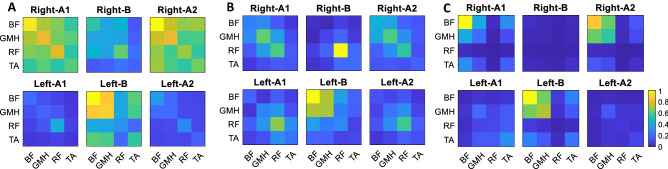
Average muscle activation (co-activation) patterns normalized to the maximum observed muscle activation in each leg separated based on movement direction, leg, and condition for (**A**) LP group, (**B**) HP group, and (**C**) the expert participant. The right leg muscles are more activated/co-activated in A1 and A2 conditions and the left muscles are more engaged in condition B. Muscle activation patterns are different along HP and LP groups. LP group tend to activate/co-activate more muscles (which lead to more bright squares in their checker plot) while the HP group exploit only one or at max two muscle of their leg similar to the expert subject (one square is super bright and the others are almost dark). This can confirm that the HP group participants have converged (learned) to a muscle activation pattern that decreases the muscle co-activation.

Condition B was designed to best interfere with the motor learning/memory of the balancing strategy acquired in condition A1. An interference would be detected if the muscle activity during A2 was significantly different from what was observed earlier in the same condition A1 (in both cases the feet point leftwards). However, we could not discern any difference in the muscle activation patterns between conditions A1 and A2. This may suggest that participants acquired a novel motor memory in condition B, which enabled them to switch between conditions A and B without interference. The parallel acquisition of two motor memories has been observed in reaching studies, but only when a unique sensorimotor cue is given to participants in each condition^26^. The different foot orientation (left in A and right in B) could have provided the necessary condition to enable our participants to learn separate motor memories in each condition without interference. This is more visible with the experienced participant (Fig. 6C) where their left leg muscle activation pattern in condition B is similar to the right leg muscle activation patterns in A1 and A2, and vice versa. Another possibility is that the duration of condition B was not sufficiently long to interfere with the motor memory of condition A.

### High and low performance groups

A positive correlation was found between the strength of the adopted ankle balance strategy and the performance of the participant. K-means clustering was applied to the number of successful trials across conditions, which divided the participants into two groups of high performance (HP) and low performance (LP) (Group HP: participants number 4, 7, 8, 9, and 10). Based on a comparison of features such as the standard deviation of the instantaneous phase, and the average distance between the ankle and hip angle trajectories, we observed that the HP group shows a stronger preference in using the ankle balance strategy in comparison to the LP group. Although the ankle strategy is dominant in HP group, as we did not record the trunk angle during the experiments, it is not clear to what extent the trunk posture contributed to control of the COG and the hoverboard plate tilt. The ankle co-activation was also lower in the HP group^24^. Interestingly, the used energy metric could also discriminate between HP and LP participants. According to Fig. 5F, LP participants had a negligible decrease in the energy, while in HP participants it dropped between 20 and 45% (participant #4 was an exception who did not exhibit a considerable reduction in energy). This may indicate that advanced hoverboard riders have a stronger tendency to maintain balance using their ankles.

The participants’ muscle recruitment patterns can be further analyzed. Figure 6A,B represent the muscle activation and co-activation computed by Eq. (3). Each tile is normalized to the maximum activation observed across muscles of each leg. As we see in these figures, the right leg’s muscles are more active in A1 and A2 conditions while the left leg’s muscles are more active in condition B. This is expected since in conditions A1 and A2, where the feet are oriented to the left, the right foot has a smaller moment arm when applying torque to the plate (see Fig. 1) so more force is required on the hoverboard’s right plate. Hence, more activity in the right leg is observed. The opposite is true for condition B. Therefore, the right leg exhibits higher muscle activation in balance control in A1 and A2 conditions while the left leg is more engaged in condition B. Furthermore, we observed that HP participants tend to activate a lower number of muscles simultaneously while the LP group recruit more muscles. This could be a determinant of lower co-activation in HP participants in comparison to the LP participants and a sign of less efficient control in the LP group.

### Kinematic measures and performance metrics

We evaluated participants’ performance based on various kinematic metrics such as maximum perpendicular deviation, maximum line crossing error, average hoverboard orientation, and line crossing angles but none of them were able to discriminate participants in a meaningful way. The underlying reason is the complexity and redundancy of hoverboard control, which could be improved through more efficient movements, but may not necessarily manifest itself as a reduction in kinematic error. A possible explanation is that the duration of the experiment was insufficient for a significant learning to occur specific to optimize the hoverboard trajectory. The sensorimotor system may have prioritized balance and more efficient use of metabolic resources rather than attempting to move the hoverboard along a straight line.

## Conclusion

This work investigated the learning of lower limb motor control and the emergent dynamic balance strategy in first-time hoverboard riders. Ten participants were asked to perform goal-oriented back and forth movements on a hoverboard in three tests, with each trial lasting for 60 s. Decreased total muscle activation, elapsed trial time, and ankle-level muscle co-activation over the performed trials indicated motor learning. The analysis of the maximum cross-correlation, phase synchrony and DTW distance showed the dominant contribution of the ankle in control of hoverboard (an ankle balance strategy). The learned ankle strategy was robust to performing the task with a different foot orientation (either pointing left or right). Further analysis suggested that the strength of the ankle strategy correlates with the performance of the participants. Additional investigation could clarify whether distinct motor memories were learned in the two conditions.

## References

[1] van der Kooij H, Jacobs R, Koopman B, Grootenboer H (1999). A multisensory integration model of human stance control. Biol. Cybern..

[2] Peterka R (2002). Sensorimotor integration in human postural control. J. Neurophysiol..

[3] Kuo AD (2005). An optimal state estimation model of sensory integration in human postural balance. J. Neural Eng..

[4] Winter DA (1995). Human balance and posture control during standing and walking. Gait Posture.

[5] Sullivan B, Harding AG, Dingley J, Gras LZ (2012). Improvements in dynamic balance using an adaptive snowboard with the Nintendo Wii. Games Heal. Res. Dev. Clin. Appl..

[6] Kinzey SJ, Armstrong CW (1998). The reliability of the star-excursion test in assessing dynamic balance. J. Orthop. Sports Phys. Ther..

[7] Davlin CD (2004). Dynamic balance in high level athletes. Percept. Mot. Ski..

[8] Rahman KA, Azaman A, Mohd Latip H, Mat Dzahir M, Balakrishnan M (2017). Comparison of tibialis anterior and gastrocnemius muscles activation on balance training devices and hoverboard. Malays. J. Fundam. Appl. Sci..

[9] Horak FB, Nashner LM (1986). Central programming of postural movements: Adaptation to altered support-surface configurations. J. Neurophysiol..

[10] Van Ooteghem K, Frank JS, Horak FB (2009). Practice-related improvements in posture control differ between young and older adults exposed to continuous, variable amplitude oscillations of the support surface. Exp. Brain Res..

[11] Gera G (2016). Postural motor learning deficits in people with ms in spatial but not temporal control of center of mass. Neurorehabilit. Neural Repair.

[12] Van Ooteghem K (2008). Compensatory postural adaptations during continuous, variable amplitude perturbations reveal generalized rather than sequence-specific learning. Exp. Brain Res..

[13] Burdet E, Osu R, Franklin DW, Milner TE, Kawato M (2001). The central nervous system stabilizes unstable dynamics by learning optimal impedance. Nature.

[14] Lee H, Ho P, Rastgaar M, Krebs HI, Hogan N (2013). Multivariable static ankle mechanical impedance with active muscles. IEEE Trans. Neural Syst. Rehabil. Eng..

[15] Amiri P, Kearney RE (2019). Ankle intrinsic stiffness changes with postural sway. J. Biomech..

[16] Huang HY, Arami A, Farkhatdinov I, Formica D, Burdet E (2020). The influence of posture, applied force and perturbation direction on hip joint viscoelasticity. IEEE Trans. Neural Syst. Rehabil. Eng..

[17] Lee H, Rouse EJ, Krebs HI (2016). Summary of human ankle mechanical impedance during walking. IEEE J. Transl. Eng. Heal. Med..

[18] Arami A, van Asseldonk E, van der Kooij H, Burdet E (2020). A clustering-based approach to identify joint impedance during walking. IEEE Trans. Neural Syst. Rehabil. Eng..

[19] Chagdes JR, Rietdyk S, Jeffrey MH, Howard NZ, Raman A (2013). Dynamic stability of a human standing on a balance board. J. Biomech..

[20] Konrad, P. The ABC of EMG: A practical introduction to kinesiological electromyography. http://www.noraxon.com/wp-content/uploads/2014/12/ABC-EMG-ISBN.pdf (2005). Accessed 09 March 2022.

[21] Marple L (1999). Computing the discrete-time “analytic” signal via fft. IEEE Trans. Signal Process..

[22] Oppenheim AV (1999). Discrete-Time Signal Processing.

[23] Sakoe H, Chiba S (1978). Dynamic programming algorithm optimization for spoken word recognition. IEEE Trans. Acoust. Speech Signal Process..

[24] Heald JB, Franklin DW, Wolpert DM (2018). Increasing muscle co-contraction speeds up internal model acquisition during dynamic motor learning. Sci. Rep..

[25] Franklin DW (2008). Cns learns stable, accurate, and efficient movements using a simple algorithm. J. Neurosci..

[26] Howard IS, Ingram JN, Franklin DW, Wolpert DM (2012). Gone in 0.6 s: The encoding of motor memories depends on recent sensorimotor states. J. Neurosci..

